# Bacteria incorporated with calcium lactate pentahydrate to improve the mortar properties and self-healing occurrence

**DOI:** 10.1038/s41598-020-74127-4

**Published:** 2020-10-21

**Authors:** Siti Khodijah Chaerun, Ridwan Syarif, Ridho Kresna Wattimena

**Affiliations:** 1 Department of Metallurgical Engineering, Faculty of Mining and Petroleum Engineering, Institut Teknologi Bandung, Ganesha 10, Bandung, 40132 Indonesia; 2 Geomicrobiology-Biomining & Biocorrosion Laboratory, Microbial Culture Collection Laboratory, Biosciences and Biotechnology Research Center (BBRC), Institut Teknologi Bandung, Ganesha 10, Bandung, 40132 Indonesia; 3 Department of Mining Engineering, Faculty of Mining and Petroleum Engineering, Institut Teknologi Bandung, Ganesha 10, Bandung, 40132 Indonesia

**Keywords:** Microbiology, Biogeochemistry, Environmental sciences, Materials science

## Abstract

Concrete can be harmful to the environment due to its high energy consumption and CO_2_ emission and also has a potential crack formation, which can promote a drop in its strength. Therefore, concrete is considered as a non-sustainable material. The mechanisms by which bacterial oxidation of organic carbon can precipitate calcite that may fill the voids and cracks on cement-based materials have been extensively investigated to prevent and heal the micro-cracks formation. Hence, this study focused on utilizing a new alkaliphilic bacterial strain indigenous to an Indonesian site, *Lysinibacillus sphaericus* strain SKC/VA-1, incorporated with calcium lactate pentahydrate, as a low-cost calcium source, with various bacterial inoculum concentrations. The bacterium was employed in this study due to its ability to adapt to basic pH, thus improving the physical properties and rejuvenating the micro-cracks. Experimentally, the addition of calcium lactate pentahydrate slightly affected the mortar properties. Likewise, bacteria-incorporated mortar exhibited an enhancement in the physical properties of mortar. The highest improvement of mechanical properties (an increase of 45% and 36% for compressive and indirect tensile strength, respectively) was achieved by the addition of calcium lactate pentahydrate incorporated with 10% v/v bacterial inoculum [about 7 × 10^7^ CFU/ml (colony-forming unit/ml)]. The self-healing took place more rapidly on bacterial mortar supplemented with calcium lactate pentahydrate than on the control specimen. XRD analysis demonstrated that the mineralogical composition of self-healing precipitates was primarily dominated by calcite (CaCO_3_), indicating the capacity of *L. sphaericus* strain SKC/VA-1 to precipitate calcite through organic carbon oxidation for self-healing the artificial crack on the mortar. To our knowledge, this is the first report on the potential utilization of the bacterium *L. sphaericus* incorporated with calcium lactate pentahydrate to increase the mortar properties, including its self-healing ability. However, further study with the water-cement ratio variation is required to investigate the possibility of using *L. sphaericus* and calcium lactate pentahydrate as an alternative method rather than reducing the water-cement ratio to enhance the mortar properties.

## Introduction

Nowadays, a high pace of population growth has led to the rapid development of buildings and infrastructure construction. Consequently, global concrete demand has enormously grown and its utilization^1,2^, causing some adverse effects on the environment due to massive cement production, which is one of the main concrete ingredients. The high energy consumption and CO_2_ global emission are the major drawback of cement production, which results in the unsustainability of concrete^3–5^. Moreover, the discontinuity and heterogeneity are generated due to sand and aggregate composition and low binder in the concrete matrix caused by a low water-cement ratio, which is indicated by micro-crack forming^6^. Consequently, the strength and durability of concrete will depress^7–9^. The continuous network of micro-cracks may affect the permeability of the concrete, reducing the concrete resistance against the ingress of aggressive substances^10^. The chemical attack from those substances, such as sulfate and chloride ions, can contribute to the deterioration of cementitious material and lead to corrosion on reinforcement concrete^11^.

An environmentally friendly biomineralization method, known as microbially induced calcium carbonate precipitation (MICCP), has been widely reported to solve these problems^12^. The most common mechanism of MICCP is through the urease activity pathway, which degrades the urea to produce carbonate ion as a by-product. The carbonate will attach to Ca^2+^ ion in the environment and precipitate calcium carbonate according to Equations S1–S2 (see “Supplementary files”)^13–16^. This mechanism has become one of the most well-known pathways due to its straightforward and easily controlled process^17^. The related previous studies reported significant increase in concrete strength parameters^9,18–22^. Another MICCP mechanism, nitrate reduction, was also investigated for precipitating calcium carbonate under anaerobic condition^23,24^. Nonetheless, this mechanism raised some drawbacks, such as excessive ammonia production and CaCl_2_ usage, which are harmful to the environment and have a detrimental effect on human health at high concentrations, yet corrosive for the reinforced concrete^9,25–27^. Hence, the other eco-friendly substrates and mechanisms are urgently required as the alternative method.

Researchers at the Delft University of Technology in the Netherlands reported that alkaliphilic bacteria, such as *Bacillus pseudofirmus* and *Bacillus cohnii*, can remediate the cracks while increasing the concrete strength by utilizing the organic carbon, such as calcium lactate, to precipitate calcium carbonate through oxidation mechanism^28–30^. The mechanism of calcium lactate oxidation is described according to Equation S3 (see “Supplementary files”). The utilization of calcium lactate as the substrate is more environmentally friendly and has an insignificant effect on the strength of concrete^28,29^. The previous study also indicated that ureolytic bacteria could enhance the strength of concrete through organic carbon oxidation. Chaurasia et al. reported that calcium lactate oxidation by *B. megaterium* and *B. pasteurii* metabolism could increase the compressive strength of concrete by 40% and 37%, respectively^31^. These bacterial concretes containing calcium lactate exhibited a slightly higher compressive strength than those containing no calcium lactate (37% for *B. megaterium* and 33% for *B. pasteurii*).

Another potential bacterium that has successfully increased concrete strength through urease activity is *Bacillus sphaericus* (renamed as *Lysinibacillus sphaericus* since 2007)^32,33^. However, this bacterium has rarely been used in organic carbon oxidation to improve concrete properties. Moreover, only a few studies reported the bacterial role in influencing cement-based material characteristics incorporated with calcium lactate pentahydrate. Hence, this study aimed to investigate the effect of the bacterium *L. sphaericus* strain SKC/VA-1 (The GenBank/EMBL/DDBJ accession number for the 16S rRNA gene sequence of strain SKC/VA-1 is MT225574) and calcium lactate pentahydrate on physical and mechanical properties of mortar. Specimens with and without calcium lactate pentahydrate in the presence of this bacterial strain were compared to determine any potential effects on mortar properties. The effect of calcium lactate pentahydrate on ordinary mortar specimens was also examined to discover the bacterial role in influencing the characteristics of mortar. In addition, self-healing occurrence was observed up to several days of wet curing as an additional evidence to convince that the oxidation of calcium lactate pentahydrate could precipitate calcium carbonate by bacterial metabolism. Expectedly, the utilization of *L. sphaericus* strain SKC/VA-1 incorporated with calcium lactate pentahydrate would be potentially applicable since it is a low-cost and more environmentally friendly alternative method for improving mortar properties.

## Results

### Porosity and water absorption measurement

**Figure 1:**
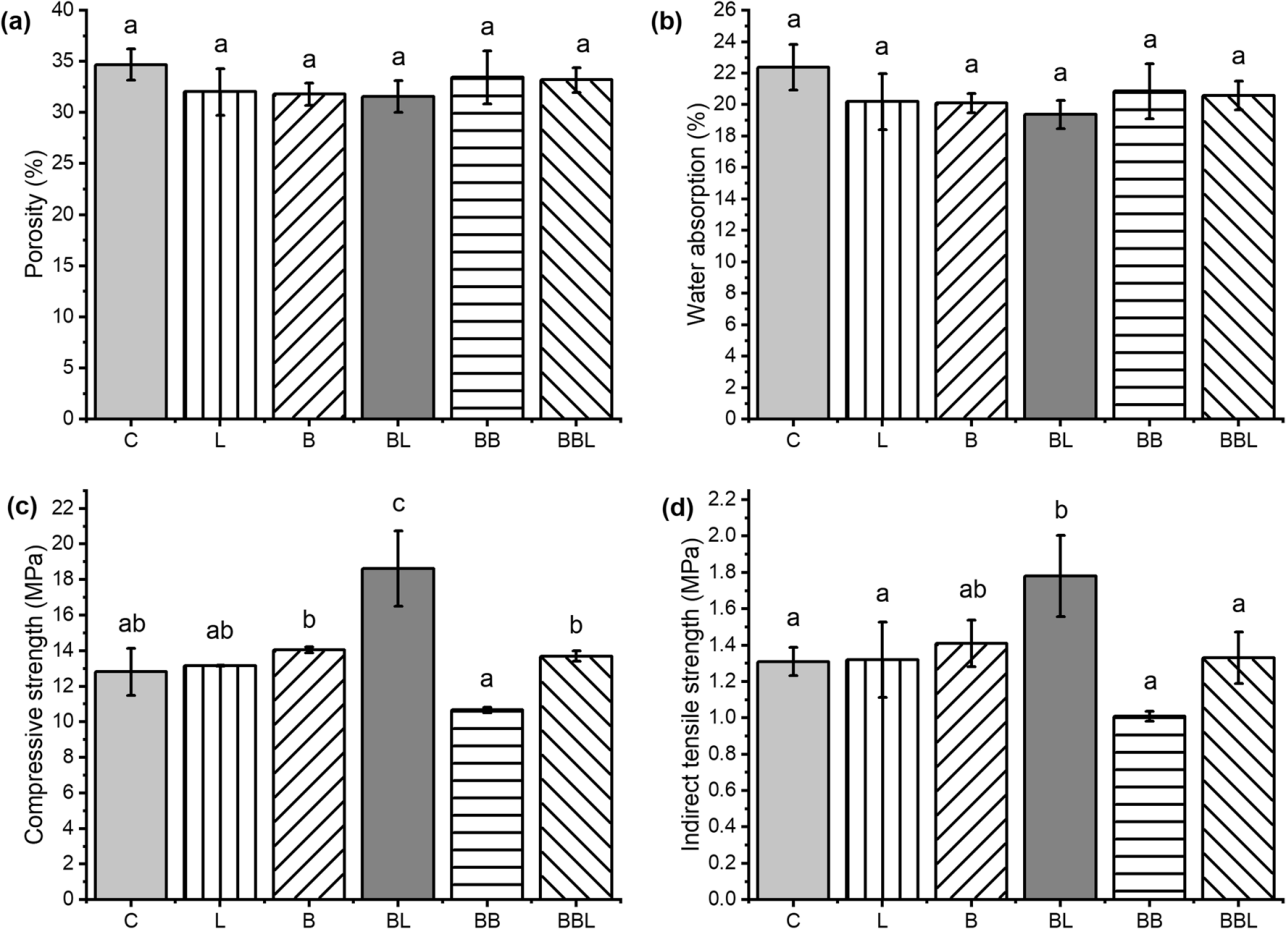
The physical and mechanical properties [porosity (**a**), water absorption (**b**), compressive strength (**c**), and indirect tensile strength (**d**)] of all mortar specimens [without both bacteria and calcium lactate (C), containing calcium lactate without bacteria (L), containing bacteria without calcium lactate (B and BB), containing both bacteria and calcium lactate (BL and BBL)] after 7-day wet curing. Data reported are the mean (bars) ± standard deviation (error bars) obtained from triplicate experiments (n = 3) with a significant level of 0.05 for ANOVA and Tukey post hoc test.

Figure 1a,b showed a slight decline in porosity and water absorption for all specimens compared to the control (C) specimen. A drop in the average porosity of each specimen (L, B, BL, BB, and BBL) was 8%, 8%, 9%, 4%, and 4%, respectively, whereas that in the average water absorption of each specimen (L, B, BL, BB, BBL) was 10%, 10%, 13%, 7%, and 8%, respectively. However, their decrease was not statistically different (p > 0.05) from the control. The reason for this behavior might be due to the high water–cement ratio used in this study (w/c = 0.5), thus generating more capillary pores and voids within the mortar matrix of all specimens.

### Compressive and indirect tensile strength test

Compared with control (C), the compressive strength of all specimens was elevated, except for the BB specimen (Fig. 1c). However, the high compressive strength of L, B, BBL specimens was not statistically different (p > 0.05), and that of the BL specimen was significantly different (p < 0.05) from that of control. The highest increase was achieved by the BL specimen, which had a remarkable increase of 45% compared with the control (C) specimen. For indirect tensile strength (the Brazilian test), as shown in Fig. 1d, an increase in strength was observed for L, B, BL, BBL specimens but not for BB specimen. Nevertheless, their strength increase was not statistically different (p > 0.05) from the control, except for the BL specimen, which had a tremendous increase of 36%.

### Crack self-healing behaviour and mineralogical observation of the precipitates

**Figure 2:**
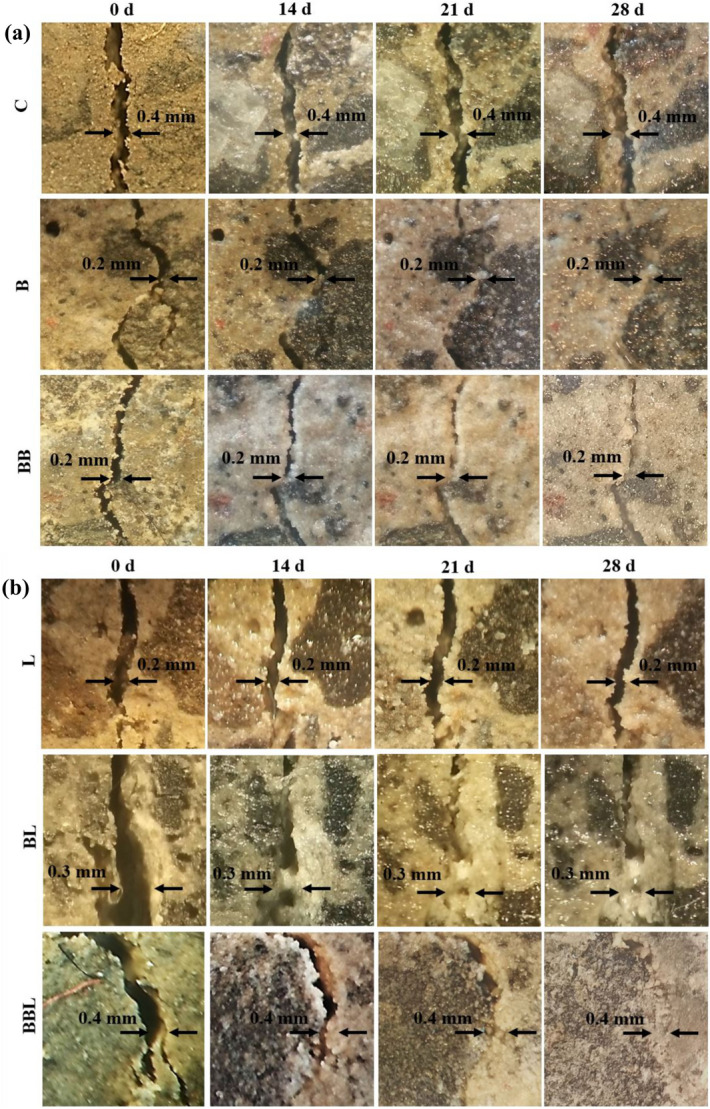
Stereomicroscopic images of the self-healing process for 28 days on the artificial crack of mortar specimens (from Brazilian test) in the absence (**a**) and presence (**b**) of calcium lactate pentahydrate. The presence of calcium lactate pentahydrate was observed to affect the calcite precipitation slightly. The bacteria incorporated with calcium lactate pentahydrate exhibited a significant crystal growth on the artificial crack, suggesting the self-healing process.

**Figure 3:**
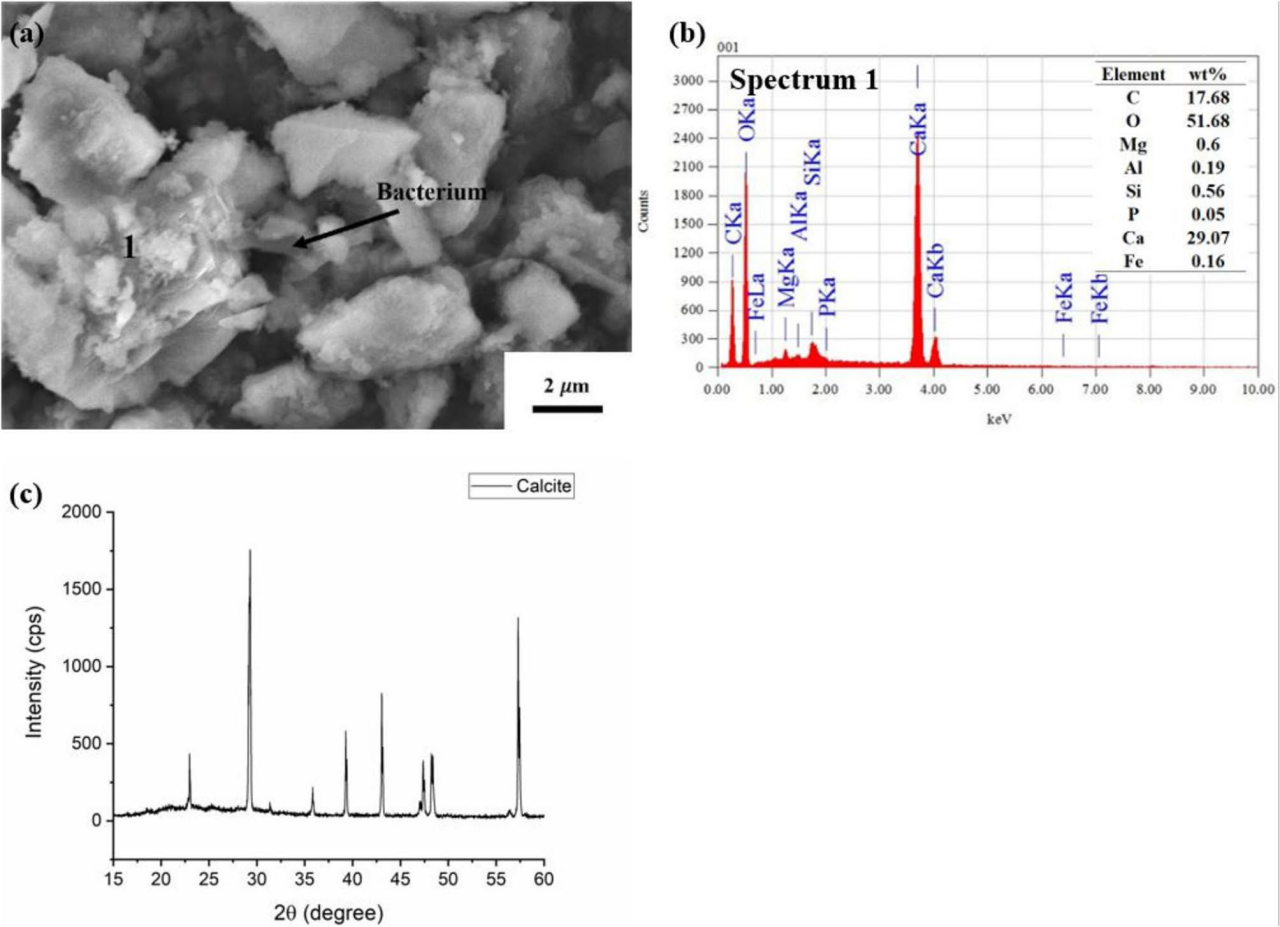
SEM image (**a**), EDS spectrum (**b**; analytical point 1), and XRD analysis (**c**) of the precipitates generated on the artificial crack of BL specimen after 28 days of the experiment, showing the evidence of calcite (CaCO_3_) precipitation by the bacterium *L. sphaericus* strain SKC/VA-1 in self-healing occurrence where the calcite mineral appeared to be surrounded by a rod-shaped bacterium (**a**).

Figure 2a,b showed the self-healing capability of mortar in the presence and absence of bacteria or calcium lactate pentahydrate (hereafter: calcium lactate). The self-healing process took place over a 28-day experiment for the specimens containing bacteria with and without calcium lactate (B, BL, BB, BBL) but not for C (without bacteria and calcium lactate) and L (containing calcium lactate but no bacteria) specimens. Specimens with higher bacterial cell concentration (BB and BBL) were observed to experience rapid crystal growth on the crack walls. Meanwhile, the specimens containing both bacteria and calcium lactate (BL and BBL) generated more precipitates than other specimens (Fig. 2b). SEM–EDS and XRD analyses were undertaken on the mineral deposits in the artificial crack to investigate the more detailed characteristics of precipitates. The mineral precipitates/deposits, which were surrounded by a rod-shaped bacterium (Fig. 3a), were identified as calcite (CaCO_3_) by EDS and XRD analyses (Fig. 3b,c). This finding suggested the self-healing ability of the mortar assisted by the bacterial activity through the formation of CaCO_3_ precipitates.

### Characterization of specimen microstructure

**Figure 4:**
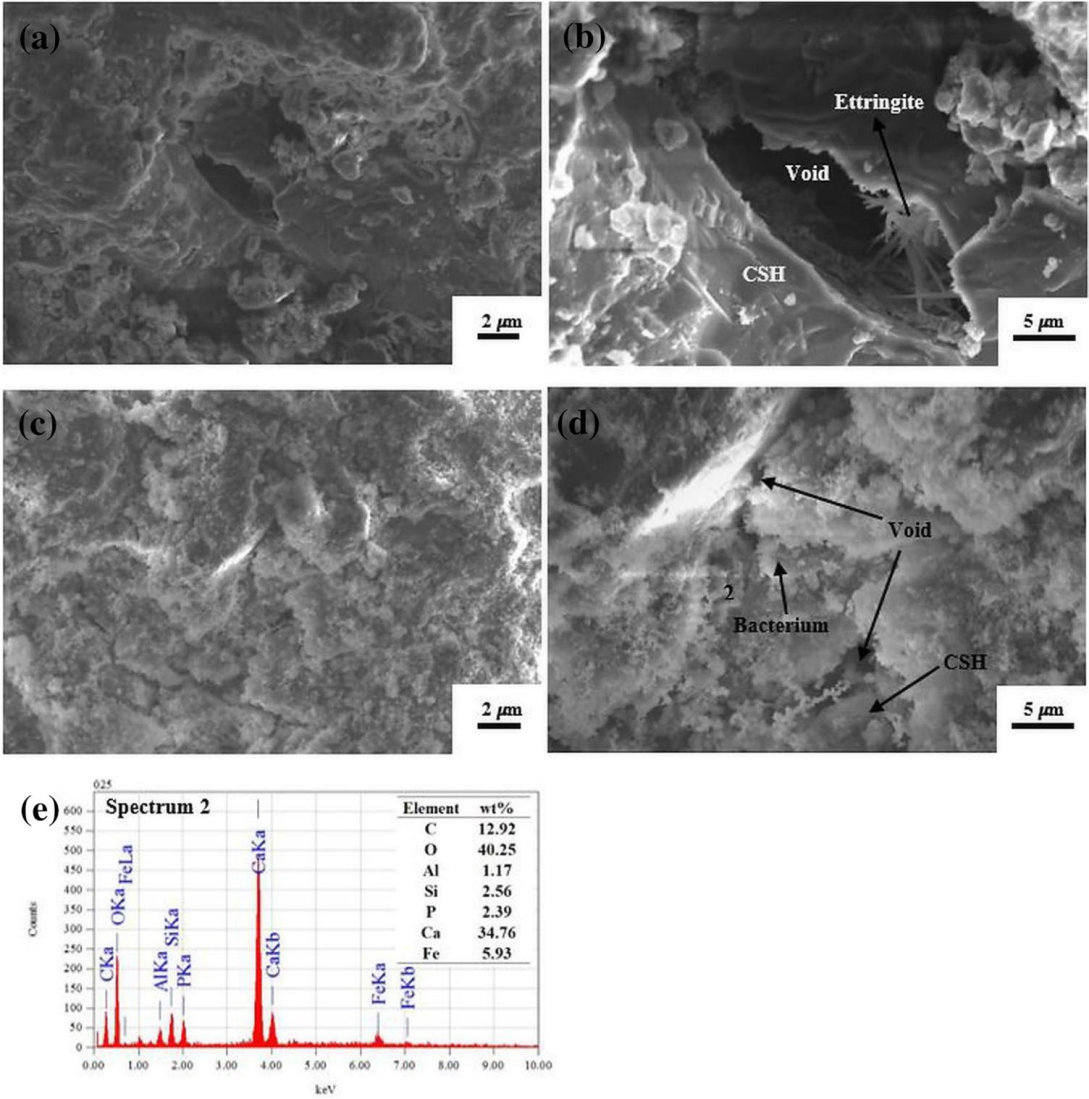
SEM images of the microstructure of C (**a**, **b**) and BL (**c**, **d**) mortar specimens. A large void in C mortar specimen was discernible (**a**, **b**), which was surrounded by calcium silicate hydrate (CSH). In contrast, the void in the BL mortar specimen was particularly clogged by mineral precipitates (**c**, **d**), which was identified by EDS analysis (analytical point 2) as calcite (**e**).

The microstructure of the specimen was visualized to investigate the calcite precipitation that filled the void within the mortar (Fig. 4). A larger void was detected in the control (C) specimen (Fig. 4a,b). On the other hand, the void in the BL specimen was filled with calcite as being identified by EDS analysis (analytical point 2) (Fig. 4e), although the void was not entirely clogged by the precipitates (Fig. 4c,d).

## Discussion

Based on the previous study, cement-based materials incorporated with *Bacillus* spp*.* are more favorable to precipitate calcium carbonate, filling the void^8^ and increasing the strength of cementitious materials^21,34^. Besides, the metabolism of those bacteria can also remediate the surface crack, well-known as self-healing concrete^10,21,28–30^. This study exhibited the benefits of using the bacterium *L. sphaericus* strain SKC/VA-1 for enhancing mortar properties. The mechanism of organic carbon oxidation through bacterial metabolism, which used calcium lactate, was evaluated in this study. The main aim of this study was to investigate the effect of calcium lactate incorporated with the bacterium *L. sphaericus* strain SKC/VA-1 and various bacterial cell concentrations on the enhancement of mortar properties and self-healing process.

We used L specimen in this study to evaluate the effects of calcium lactate on the physical and mechanical properties of the mortar specimens. Experimental evidence revealed that calcium lactate did not significantly weaken the physical and mechanical properties of the mortar, which was in line with the previous study. Ca^2+^ ion from calcium lactate likely contributed to the generation of calcium silicate hydrate, calcium hydroxide, and calcium carbonate on cement paste through hydration and carbonation reaction. Consequently, the mortar matrix shifted to solid-state, therefore reducing the void existence^29,31^. However, further research is necessary to investigate various calcium lactate concentrations on porosity and water absorption.

Concerning bacterial mortar specimens without calcium lactate (BB specimen), the declined mortar strength might be caused by the deficient precipitation of calcium carbonate inside the mortar matrix under bacterial metabolisms at 20% v/v of bacterial inoculum in spite of the most precipitation for self-healing on the artificial crack for 28 days. This phenomenon might occur due to the different optimum bacterial concentrations, as reported by the previous studies^34,35^. In comparison, the bacterial mortar specimens with calcium lactate (BL specimen) exhibited the highest increase in mortar strength (Fig. 1c,d), which agreed with the previous study by the scientists of the Delft University of Technology in the Netherlands, reporting the calcium carbonate precipitation through calcium lactate (see “Supplementary files”; Equation S3)^28–30^.

Moreover, CO_2_ produced from the reaction could be interconverted to HCO_3_^−^ since the specimens were cured in water. Equilibrium reaction in HCO_3_^−^ would produce CO_3_^2−^ and could precipitate calcium carbonate due to Ca^2+^ existence. Consequently, calcium carbonate production would be more accumulated and could have a cumulative effect on the enhancement of mortar properties. This potential reaction has been called as carbonic anhydrase activity according to the Equations S4–S6 (see “Supplementary files”)^36,37^. Besides, the carbonation reaction on the mortar mixture also converted portlandite minerals (Ca(OH)_2_) into calcium carbonate (CaCO_3_) according to Equation S7 (see “Supplementary files”), which contributed to the enhancement of the cementitious material characteristics^38^.

Calcium carbonate produced based on the aforementioned reactions could fill the pore and compact the cement-based materials, resulting in strength enhancement^31,34,39^. This product could be a useful filling material that can bind tiny aggregates within the mortar matrix. Even though the precipitates might not entirely clog the void, the calcium carbonate deposits have been essential enough to increase the mortar properties. Moreover, this product might be randomly deposited on both inside and outside the mortar. For example, the self-healing precipitates on the surface crack of BL specimen was less than BBL specimen, although its increase in mortar properties was the most optimum among the mortar specimens. This behavior was probably due to the localization of calcium carbonate precipitate, which was higher on the surface than other specimens^34^.

Therefore, the current study was the first report, demonstrating *L. sphaericus* through calcium lactate oxidation for producing calcium carbonate. In this study, *L. sphaericus* could adapt to alkaline environments such as those at a high pH of cementitious materials, which coincided with the previous study^33^. Since it was successfully performed in the mortar, this approach could be beneficial for other cement-based materials. This study may also suggest that the optimum mixture of the bacterial cell concentration of *L. sphaericus* and calcium lactate may improve mortar strength and lower the water-cement ratio. However, the further studies are needed to optimize the bacterial mortar parameters to obtain the best performance in precipitating calcium carbonate, including (1) varying water-cement ratios and (2) the application of the bacterium and calcium lactate to a field-scale concrete construction or even shotcrete for underground construction and mining applications.

## Conclusions

The present study exhibited the potential of a locally isolated alkaliphilic bacterium, *L. sphaericus* strain SKC/VA-1, and calcium lactate pentahydrate as substrates on the mortar mixture to enhance the properties of mortar and self heal its artificial crack. The mixture of bacterial cell concentration (10% v/v) and calcium lactate pentahydrate (0.5% w/w of cement) could be recommended to be applied in bacterial concrete since such a mixture exhibited the optimum improvement of mortar properties, although the self-healing phenomenon on its artificial crack showed the most optimum condition by incorporating 20% v/v of bacterial inoculum to the mortar. Under this condition, the enhanced mortar quality with self-healing ability can be produced while cutting-off the maintenance cost. This strategy can be used as a candidate for innovative technology to make the construction more environmentally friendly and sustainable. Nevertheless, further study with varying water-cement ratios is required to investigate the potential of using the bacterium *L. sphaericus* strain SKC/VA-1 and calcium lactate pentahydrate as an alternative method rather than reducing the water-cement ratio to increase the mortar properties.

## Materials and methods

### Microorganisms

A local bacterial species isolated from a mixture sample containing crude oil and rust deposits of petroleum pipelines collected in Sukabumi, West Java, Indonesia, *L. sphaericus* strain SKC/VA-1*,* was used in this study. As described previously, this species was grown in a modified culture medium containing 1.5 g/L nutrient broth (Oxoid) and 3.2 mM calcium lactate pentahydrate (CaC_6_H_10_O_6_·5H_2_O; purchased from a local chemicals store, Bandung, West Java, Indonesia)^40^. The medium was then sterilized by autoclaving at 121 °C for 15 min, followed by inoculation of 10% v/v bacterial inoculum at a cell concentration of 7 × 10^7^ CFU/ml (colony-forming unit/ml), finally incubated on a rotary shaker of 180 rpm at room temperature (25 °C) for 48 h. The bacterial culture was kept in an incubator for later usage for specimen preparation.

### Preparation of mortar specimens

Cylindrical mortar specimens were prepared based on a mixture design (Table 1). Ordinary Portland Cement (Type 1, SNI 15-2049-2004)^41^, fine aggregate (0–2 mm and 2–5 mm), and tap water were used as the basic components of the mortar specimens. The control specimen containing no bacterium and calcium lactate was designated C (Table 1). The bacterial culture at the concentration of 7 × 10^7^ CFU/ml (colony-forming unit/ml) was incorporated at 10% and 20% in a liquid state as a water replacement within the mixture. Calcium lactate pentahydrate (0.5% w/w of cement) was used as the substrate for bacterial nutrients according to Jonkers et al.^29^. Calcium lactate pentahydrate has good solubility in water (solubility of around 6–7 g/100 g water at room temperature)^42^, thereby being used in the organic carbon oxidation pathway for precipitating calcium carbonate. However, to our knowledge, the hydrates in this compound do not significantly affect the water content in organic carbon oxidation reactions, which are fully described in more detail in Equations S8–S15 (see “Supplementary files”). In addition, the fabrication of all specimens was described in “Supplementary files”.

**Table 1:** The experimental methodology consisted of six different mortar mixture components.

Component	Specimen					
	C	L	B	BL	BB	BBL
Cement (g)	600	600	600	600	600	600
Fine aggregate; 2–5 mm (g)	1080	1080	1080	1080	1080	1080
Fine aggregate; 0–2 mm (g)	720	720	720	720	720	720
Tap water (cm^3^)	300	300	270	270	240	240
Bacteria (cm^3^)	0	0	30	30	60	60
Calcium lactate pentahydrate (g)	0	3	0	3	0	3

### Porosity and water absorption

Porosity and water absorption measurements were carried out on specimens of 4.5 cm in diameter and 3 cm long. Firstly, all specimens were cured by water immersion for 7 days, withdrawn, dried in the oven at 105 °C for 24 h, and weighed as the oven-dried mass. Subsequently, the specimens were further immersed in a water tank for 48 h, withdrawn from the water, wiped, and weighed for each saturated surface-dry mass. Finally, specimens were wire-tied and submerged in water for measuring the apparent mass in water. Determination of porosity^21^ and water absorption^43^ was based on Equations S16–S17 (see “Supplementary files”).

### Compressive and indirect tensile strength test

Compressive and indirect tensile strength (Brazillian) tests were carried out using the ISRM Suggested Method on specimens of 9 cm and 2.25 cm in height, which had the same diameter of 4.5 cm^44,45^. After being cut at both ends, the specimens were immersed in water for 7 days. The tests were conducted using a computer-controlled servo-hydraulic concrete compression testing machine (Hung Ta, HT-8391) with the loading rates given to each compressive and tensile strength of 6000 N/min and 2000 N/min, respectively. The determination of compressive strength and indirect tensile strength (Brazilian test) was according to Equations S18–S19 (see “Supplementary files”).

### Self-healing and microstructure observations

The self-healing process was visually observed using a trinocular stereomicroscope (SMZ-2T, Nikon) to investigate bacterial healing due to calcium carbonate precipitation on the surface crack of the specimen. The specimens with artificial crack from the Brazilian tensile strength test were used in this observation. Subsequently, the precipitates were characterized mineralogically and microscopically by using XRD and SEM–EDS. A detailed description of self-healing observation, XRD examination of the precipitates, and the preparation of specimens for SEM–EDS observation was given in “Supplementary files”. In addition, SEM–EDS (JEOL JSM-J6510 A) observation was also conducted to characterize the microstructure and elemental composition of the mortar specimens, particularly inside the mortar matrix. In comparison, the mortar specimens with and without bacterial incorporation were also observed. The thin fragments were taken from specimens, then prepared for SEM–EDS observation (see “Supplementary files”).

### Statistical analysis

The experiments of physical and mechanical properties were conducted in triplicate. The data are presented as the arithmetic mean ± standard deviation of the mean. One-way ANOVA followed with Tukey post hoc test with a significant level (α) of 95% was used to identify the significant differences between each type of the specimens related to physical and mechanical properties. Meanwhile, the Pearson test was carried out for determining the correlation between the physical and mechanical properties. The relationship between physical and mechanical properties was described in “Supplementary files”. Statistical analysis was undertaken using SPSS version 25 (IBM SPSS Inc., Chicago, 1 L, USA).

## Supplementary information

Supplementary Information
