# Supplementary material for: Bacteria incorporated with calcium lactate pentahydrate to improve the mortar properties and self-healing occurrence
Source: Sci Rep. 2020 Oct 21;10:17873. doi: 10.1038/s41598-020-74127-4 (PMC7578004; doi:10.1038/s41598-020-74127-4)
Supplement: Supplementary file 1 — Supplementary Information [file 41598_2020_74127_MOESM1_ESM.doc]

**Supplementary Material**

**Bacteria incorporated with calcium lactate pentahydrate to improve the mortar properties and self-healing occurrence**

Siti Khodijah Chaerun^1,2^, Ridwan Syarif^2,3^, Ridho Kresna Wattimena^3^

^1^Department of Metallurgical Engineering, Faculty of Mining and Petroleum Engineering, Institut Teknologi Bandung, Ganesha 10, Bandung 40132, Indonesia

^2^Geomicrobiology-Biomining & Biocorrosion Laboratory, Microbial Culture Collection Laboratory, Biosciences and Biotechnology Research Center (BBRC), Institut Teknologi Bandung, Ganesha 10, Bandung 40132, Indonesia

^3^Department of Mining Engineering, Faculty of Mining and Petroleum Engineering, Institut Teknologi Bandung, Ganesha 10, Bandung 40132, Indonesia

Correspondence and requests for materials should be addressed to SKC (skchaerun@gmail.com; skchaerun@metallurgy.itb.ac.id) or RS (ridwantedjokusumo@gmail.com)

**______________________________________________________________________________**

**Microbially induced calcium carbonate precipitation through urease activity:**


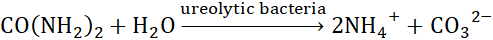
 (Equation S1)


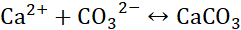
 (Equation S2)

**______________________________________________________________________________**

**Microbially induced calcium carbonate precipitation through calcium lactate oxidation:**


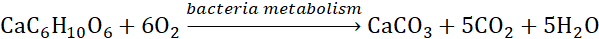
 (Equation S3)

**______________________________________________________________________________**

**Microbially induced calcium carbonate precipitation through carbonic anhydrase activity:**


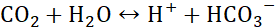
 (Equation S4)


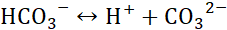
 (Equation S5)


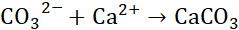
 (Equation S6)

**______________________________________________________________________________**

**Carbonation reaction of portlandite minerals (Ca(OH)_2_) that chemically precipitate calcium carbonate (CaCO_3_):**


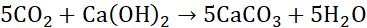
 (Equation S7)

**______________________________________________________________________________**

**Fabrication of all mortar specimens**

The mortar specimens were fabricated manually in PVC pipes with a height of 17 cm and an inner diameter of 4.5 cm. The mixtures were poured into 3 layers in the molds and rodded (32 times for each layer) by a tamping rod. Subsequently, they were immediately covered with a plastic wrap and then stored at room temperature. After 24 h, the specimens were withdrawn from the molds and cut into the required size before each test.

**______________________________________________________________________________**

**The reason why hydrates do not have any significant effects on water content can be explained as follows:**


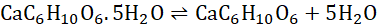
 (Equation S8)

Since the calcium lactate pentahydrate content used in this study was 0.05% of cement weight shown in Table 1 (3 g), its moles could be determined as follows:


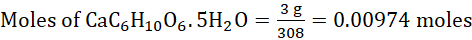
 (Equation S9)

By equilibrium constant, moles of the calcium lactate are the same as calcium lactate pentahydrate; therefore, its mass can be roughly calculated as follows:


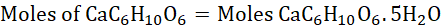
 (Equation S10)


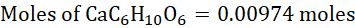
 (Equation S11)


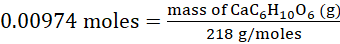
 (Equation S12)


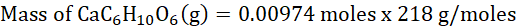
 (Equation S13)


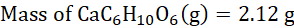
 (Equation S14)

This calculation roughly interprets that the mass of hydrates is 3 – 2.12 = 0.88 g. By knowing the density of water (1 g/ml), this value is deficient compared with the amount of water used in the mortar mixture (300 ml) in which mass percentage of hydrates can be calculated as follows:

= 0.88/300 x 100%= 0.293% (Equation S15)

As a result, the hydrates in calcium lactate pentahydrate do not interfere with the water content, which is negligible.

**______________________________________________________________________________**

**Determination of porosity and water absorption:**


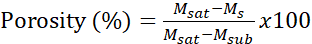
 (Equation S16)


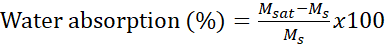
 (Equation S17)

Where *M_sat_* is the saturated surface-dry mass (g), *M_s_* is the oven-dry mass (g), and *M_sub_* is the saturated-submerged mass (g).

**______________________________________________________________________________**

**Determination of compressive strength and indirect tensile strength (Brazilian test):**


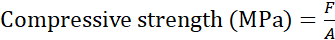
 (Equation S18)


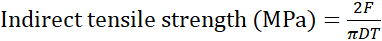
 (Equation S19)

Where *F* is a compressive force (MN), *A* is the cross-section area of the specimen (m), *D* is the diameter of the specimen (m^2^), and *T* is the thickness of the specimen (m).

**______________________________________________________________________________**

**Relationship between physical and mechanical properties**. According to Table S1 (see Supplementary files), a strong correlation existed between porosity and water absorption (r = 0.966), where the relationship between the two variables was statistically significant at α = 0.01 (P<0.01). A statistically significant linear relationship (r = 0.78, P<0.01) also existed between the compressive and indirect tensile strength, describing a correlation coefficient of r = 0.78 as a strong or high correlation. However, no correlation existed between physical and mechanical properties (P>0.01), although there was a small tendency of the inverse relationship between physical and mechanical properties as indicated by the negative value of the Pearson correlation. For instance, as shown in Fig. 1, as the porosity and water absorption in bacterial mortar specimens decreased, the compressive and indirect tensile strength in bacterial mortar specimens increased.

**______________________________________________________________________________**

**Table S1** Correlation between physical and mechanical properties of all mortar specimens

| **Correlation** | | | | | |
| --- | --- | --- | --- | --- | --- |
|  | | Porosity | Compressive strength | Water absorption | Tensile strength |
| Porosity | Pearson Correlation (r) | 1 | -0.366 | 0.966^**^ | -0.207 |
|  | Sig. (2-tailed) (P) |  | 0.136 | 0.000 | 0.410 |
|  | N | 18 | 18 | 18 | 18 |
| Compressive strength | Pearson Correlation (r) | -0.366 | 1 | -0.412 | 0.780^**^ |
|  | Sig. (2-tailed) (P) | 0.136 |  | 0.089 | 0.000 |
|  | N | 18 | 18 | 18 | 18 |
| Water absorption | Pearson Correlation (r) | 0.966^**^ | -0.412 | 1 | -0.232 |
|  | Sig. (2-tailed) (P) | 0.000 | 0.089 |  | 0.355 |
|  | N | 18 | 18 | 18 | 18 |
| Tensile strength | Pearson Correlation (r) | -0.207 | 0.780^**^ | -0.232 | 1 |
|  | Sig. (2-tailed) (P) | 0.410 | 0.000 | 0.355 |  |
|  | N | 18 | 18 | 18 | 18 |

**______________________________________________________________________________**

**The self-healing observation and XRD analysis of the precipitates**

The specimens with artificial crack from the Brazilian tensile strength test were prepared. The initial crack at the size ranging from 0.1 to 0.5 mm on each specimen was observed to trace the self-healing process. All specimens were then immersed in water for 28 d. The specimens were withdrawn, dried up, and observed for the self-healing process periodically on 0, 14, 21, and 28 d. The precipitates on the artificial crack were scraped off carefully and dried up at room temperature before XRD analysis. The mineralogical composition of the dried precipitates was determined by X-ray powder diffraction (XRD) operated with a Bruker D8 Advance diffractometer (Cu-Kα radiation, 15^o^ to 60^o^) with a step size of 0.02^o^ (19.10 s/step).

**______________________________________________________________________________**

**Preparation of cement mortar specimens for scanning electron microscope-energy-dispersive X-ray spectroscopy (SEM-EDS) observation**

The cement mortar specimens were chemically fixed by 2.5% glutaraldehyde for 24 h, washed 3 times by phosphate buffer, dehydrated by a graded acetone series (25%, 50%, 75%, 100% for 15 min, 15 min, 15 min, and 24 h, respectively), and carbon-coated before SEM-EDS observation.
